# Alternative splicing and translation play important roles in hypoxic germination in rice

**DOI:** 10.1093/jxb/ery393

**Published:** 2018-12-10

**Authors:** Mo-Xian Chen, Fu-Yuan Zhu, Feng-Zhu Wang, Neng-Hui Ye, Bei Gao, Xi Chen, Shan-Shan Zhao, Tao Fan, Yun-Ying Cao, Tie-Yuan Liu, Ze-Zhuo Su, Li-Juan Xie, Qi-Juan Hu, Hui-Jie Wu, Shi Xiao, Jianhua Zhang, Ying-Gao Liu

**Affiliations:** 1 State Key Laboratory of Crop Biology, College of Life Science, Shandong Agricultural University, Taian, Shandong, China; 2 Co-Innovation Center for Sustainable Forestry in Southern China, College of Biology and the Environment, Nanjing Forestry University, Nanjing, China; 3 Shenzhen Research Institute, The Chinese University of Hong Kong, Shenzhen, China; 4 State Key Laboratory of Biocontrol and Guangdong Provincial Key Laboratory of Plant Resources, School of Life Sciences, Sun Yat-sen University, Guangzhou, China; 5 Southern Regional Collaborative Innovation Center for Grain and Oil Crops in China, Hunan Agricultural University, Changsha, China; 6 School of Life Sciences, The Chinese University of Hong Kong, Shatin, Hong Kong; 7 SpecAlly Life Technology Co., Ltd, Wuhan, China; 8 College of Life Sciences, Nantong University, Nantong, Jiangsu, China; 9 Department of Biology, Hong Kong Baptist University, and State Key Laboratory of Agrobiotechnology, The Chinese University of Hong Kong, Shatin, Hong Kong

**Keywords:** Alternative splicing, hypoxia, *Oryza sativa*, proteogenomics, seed germination, splicing factor, translation initiation

## Abstract

Post-transcriptional mechanisms (PTMs), including alternative splicing (AS) and alternative translation initiation (ATI), may explain the diversity of proteins involved in plant development and stress responses. Transcriptional regulation is important during the hypoxic germination of rice seeds, but the potential roles of PTMs in this process have not been characterized. We used a combination of proteomics and RNA sequencing to discover how AS and ATI contribute to plant responses to hypoxia. In total, 10 253 intron-containing genes were identified. Of these, ~1741 differentially expressed AS (DAS) events from 811 genes were identified in hypoxia-treated seeds compared with controls. Over 95% of these were not present in the list of differentially expressed genes. In particular, regulatory pathways such as the spliceosome, ribosome, endoplasmic reticulum protein processing and export, proteasome, phagosome, oxidative phosphorylation, and mRNA surveillance showed substantial AS changes under hypoxia, suggesting that AS responses are largely independent of transcriptional regulation. Considerable AS changes were identified, including the preferential usage of some non-conventional splice sites and enrichment of splicing factors in the DAS data sets. Taken together, these results not only demonstrate that AS and ATI function during hypoxic germination but they have also allowed the identification of numerous novel proteins/peptides produced via ATI.

## Teaser

Using RNA sequencing and qualitative and quantitative proteomics, we unravel alternative spliced isoforms and new ‘frame’ proteins during hypoxic germination in rice.

## Introduction

Rice is a staple food that provides dietary nutrition for >2 billion people around the world (Yang and Zhang, 2006). In addition, rice is a model monocot plant used in modern research. Rice has been reported to have the ability to survive periods of submergence from seed germination to adult plants (Atwell *et al.*, 2015). In particular, it has been documented as one of the few species that can germinate under anoxia by elongating its coleoptile to reach the water surface (Miro and Ismail, 2013). This adaptation to oxygen deprivation caused by flooding can be used as a model to study molecular mechanisms in response to hypoxic or anoxic conditions. Flooding is becoming one of the most severe abiotic stresses worldwide (Sasidharan *et al.*, 2017). As the primary stresses of flooding, hypoxia and anoxia have received much attention in the past decade. Under normal oxygen concentrations, oxygen gradients have been reported in dense plant organs, including seeds, fruits, and tubers (Sasidharan *et al.*, 2017). Thus, studying the molecular mechanisms during hypoxic or anoxic conditions may facilitate an understanding of the function of O_2_ molecules in both stress response and plant development. In recent years, an N-end rule protein degradation pathway has been proposed to be an important oxygen-sensing mechanism in Arabidopsis (Gibbs *et al.*, 2011; Licausi *et al.*, 2011). Its downstream components, plant ethylene-responsive transcription factors, are affected by this pathway to activate or deactivate their target genes in response to hypoxia (Weits *et al.*, 2014; Giuntoli *et al.*, 2017). Increasing numbers of loci involved in flooding responses have been characterized, including those in lipid signalling (Xie *et al.*, 2015), jasmonic acid and antioxidant pathways (Yuan *et al.*, 2017), protein kinase (Chang *et al.*, 2012), and transcription factors (Giuntoli *et al.*, 2017). However, few studies are related to functional characterization of rice genes during flooding germination. In addition to the classical CIPK15–SnRK1A–MYBS1-mediated sugar-sensing pathway (Lu *et al.*, 2007; Lee *et al.*, 2009), a mitochondrion-localized protein (OsB12D1) has been reported to enhance flooding tolerance in rice germination and subsequent seedling growth (He *et al.*, 2014). In addition, a rice trehalose-6-phosphate (T6P) phosphatase (OsTPP7) gene has been proposed to increase sink strength in response to flooding germination (Kretzschmar *et al.*, 2015).

With the development of large profiling techniques, considerable efforts have been made to study the global changes in transcripts, protein abundance, and metabolic variation during rice hypoxic germination (Lasanthi-Kudahettige *et al.*, 2007; Narsai *et al.*, 2009, 2011, 2015; Sadiq *et al.*, 2011; Hsu and Tung, 2017). Although comparative analysis indicates that part of the hypoxic-responsive pathways is conserved among several species (Narsai *et al.*, 2011), the mechanism of adult flooding tolerance may be greatly different from that of seed flooding tolerance (Lu *et al.*, 2007; Lee *et al.*, 2009). Furthermore, recent RNA sequencing (RNA-seq) analysis using eight Arabidopsis ecotypes suggests that alternative splicing (AS) could be another pivotal factor involved in hypoxic responses (van Veen *et al.*, 2016). AS results from post-transcriptional control of eukaryotic intron-containing genes. Recent advances reveal that >95% of genes have splicing isoforms in mammals (Eckardt, 2013). Two major types of splicing complex have been documented that can determine the splicing site sequences. One is the U2 complex, which can splice at a 5'-GT–AG-3' exon–intron junction. The other is called the U12 complex and is able to utilize 5'-AT–AC-3' as a splicing junction (Zdraviko *et al.*, 2005; Will and Luhrmann, 2011). AS from multiexonic genes has been regarded as a potential way to increase plant genome coding ability (James *et al.*, 2012; Rühl *et al*., 2012; Chang *et al.*, 2014; Feng *et al.*, 2015). In addition to AS, another type of post-transcriptional regulation defined as alternative translation initiation (ATI) is involved in contributing to protein diversity (Sonenberg and Hinnebusch, 2009). Recent identification of translation initiation sites using advanced technology such as ribosome sequencing and MS-based proteomics reveals that a large number of these sites are not conventional AUG sequences (Sonenberg and Hinnebusch, 2009; Ingolia *et al.*, 2011; Lee *et al.*, 2012). In comparison with AS regulation (James *et al.*, 2012; Rühl *et al*., 2012; Chang *et al.*, 2014; Feng *et al.*, 2015; Wang *et al.*, 2015; Zhan *et al.*, 2015; Thatcher *et al.*, 2016), the function of ATI has seldom been reported in plants (de Klerk and ’t Hoen, 2015). The above techniques have demonstrated that the eukaryotic genome has the ability to encode short peptides, including upstream ORFs (uORFs) and other small ORFs (sORFs), that are located in previously marked non-coding regions of the genome (Tavormina *et al.*, 2015). Several peptides have been characterized as showing crucial roles in regulating plant development and stress responses (Simon and Dresselhaus, 2015; Tameshige *et al.*, 2016).

In summary, although stress-induced genome-wide AS changes have been extensively documented in various plant species (Yang *et al.*, 2015; Thatcher *et al.*, 2016; van Veen *et al.*, 2016; Fesenko *et al.*, 2017), the quantification of corresponding alternatively spliced isoforms at the protein level has seldom been reported. In this study, a parallel RNA-seq and proteomic approach defined as proteogenomics has been applied to achieve integrative analysis using both transcriptome and proteome data. Given our previous experience in ABA-regulated AS analysis (Zhu *et al.*, 2017), we further improved our analytical pipeline for the determination of AS- and ATI-induced genome coding ability. The results from this study further expand our understanding of genome coding ability in rice seeds, suggesting an underlying regulatory network resulting from AS and ATI during rice germination under hypoxic conditions. Understanding this hidden network may facilitate the agricultural production of rice that is suitable for direct seeding systems and provide guidelines for improving hypoxic tolerance in other crop species.

## Materials and methods

### Plant material, growth conditions, and hypoxic treatment

Seeds of *Oryza sativa* (Nipponbare) were surface-sterilized with 75% ethanol for 1 min and with 20% bleach (Clorox, ~6.25% NaClO) supplemented with 0.1% Tween-20 for 30 min before experiments. Sterilized seeds (~30–50 individuals) were imbibed on Petri dishes with three layers of filter paper and 7 ml of distilled water, and then were immediately transferred to air control or hypoxia conditions under complete darkness. The hypoxia treatment was carried out using the Whitley H35 Hypoxystation (Don Whitley Scientific Limited, UK) with a 3% O_2_ level at 28 °C, whereas air control samples were treated under the same condition in a growth chamber with air O_2_ level. Seed samples (three Petri dishes per treatment) were harvested at 6 h after treatments. Subsequently, all three biological replicates of each treatment (~30–50 seeds in one Petri dish were pooled as one biological replicate) were used as primary materials for further transcriptomic and proteomic analysis.

### Hypoxia phenotyping and measurement of physiological parameters

For phenotypic comparison, hypoxia treatments of rice seeds were conducted at 0.1% or 3% O_2_ against the air control group for 7 d. Rice seeds (~30–50 individuals) were imbibed on Petri dishes and immediately subjected to hypoxia treatment. Among these, three Petri dishes were used for the calculation of germination percentage for five consecutive days. Three Petri dishes from each treatment group were used for subsequent measurement of coleoptile length and fresh weight on day 7. Photos were also taken on day 7.

### Measurement of biochemical indicators

Biochemical indicators were measured using corresponding assay kits from Solarbio Life Sciences (Beijing, China) following the manufacturer’s protocols. In detail, the Starch Content Assay Kit (Solarbio, Cat#BC0700), α-Amylase Assay Kit (Solarbio, Cat#BC0615), Hydrogen Peroxide Assay Kit (Solarbio, Cat#BC3595), and Proline Content Assay Kit (Solarbio, Cat#BC0290) were obtained for subsequent spectrophotometric measurements.

### Rice seed RNA extraction and RNA sequencing

Rice seed total RNAs were ground in liquid nitrogen and extracted using a Plant RNeasy Mini Kit (Qiagen, Germany) according to the manufacturer’s instructions. RNA-seq experiments were conducted as previously described with minor modifications (Zhu *et al.*, 2017). The resulting cDNA library constructed from rice seed RNA samples (Air_6 h and Hypoxia_6 h) were used for paired-end (2 × 125 bp) sequencing on an Illumina HiSeq 4000 platform by Annoroad Gene Technology Co. Ltd (Beijing, China). Three replicates for each sample were trimmed to obtain clean reads for subsequent analysis (see Supplementary Table S1 at *JXB* online).

### Analysis of RNA sequencing and proteomic data

The rice (Nipponbare) reference genome annotation file (Oryza_sativa.IRGSP-1.0.32) was downloaded from the Ensembl website (http://www.ensembl.org/index.html). Mapping of clean reads and subsequent bioinformatic analysis were as described previously (Zhu *et al.*, 2017). The analytical pipeline is summarized in Supplementary Fig. S1. As mentioned previously (Zhu *et al.*, 2017), significant changes in differentially expressed genes (DEGs) (Supplementary Table S2) and differentially expressed alternative splicing (DAS) events (Supplementary Table S3) were determined as log_2_FC >2 and *q*-value (false discovery rate, FDR <5%). Identification and quantification of AS events were conducted by using the software ASprofile (http://ccb.jhu.edu/software/ASprofile) (Florea *et al.*, 2013). Splicing junctions reported in this study were generated by default settings of TopHat v2.1 aligner. The AS events with no expression values were filtered out before subsequent analysis (Zhu *et al.*, 2017). Gene Ontology (GO) analysis (http://geneontology.org/) and Kyoto Encyclopedia of Genes and Genomes (KEGG; http://www.kegg.jp/) enrichment classification were carried out using both DEG and DAS data sets. Heatmaps were generated using the BAR HeatMapperPlus tool (http://bar.utoronto.ca/ntools/cgi-bin/ntools_heatmapper_plus.cgi). The splicing sites conservation analysis was performed using WebLogo v3 (http://weblogo.threeplusone.com/) (Crooks *et al.*, 2004). Prediction of protein subcellular localization was carried out by using online server WoLF PSORT (https://wolfpsort.hgc.jp/).

### Total protein extraction, digestion, and qualitative identification

Total protein of rice seeds was extracted and digested as described previously (Chen *et al.*, 2014) with minor modifications. In general, ~5 g of rice seed tissues of each sample were ground in liquid nitrogen for subsequent proteomic analysis. The precipitated protein pellets were digested by trypsin and desalted using a Sep-Pak C_18_ column (Waters). The resulting peptides were then separated and characterized in a TripleTOF 5600^+^ (AB SCIEX) splitless Ultra 1D Plus (Eksigent) system (Andrews *et al.*, 2011).

### Peptide dimethyl labelling and quantitative proteomics

The quantitative proteomics were conducted as described previously with minor modifications (Zhou *et al.*, 2015). Digested peptides were dissolved with 0.1 M sodium acetate (pH ~6, best below 6) (i.e. 500 μg peptides per 0.25 ml of sodium acetate). Either 4% formaldehyde or formaldehyde-d2 (40 μl per 500 μg of peptides) was added and mixed. Then, 40 μl per 500 μg of peptides of 0.6 M NaBH_3_(CN) were added. The solution mixture was shaken for 0.5 h. Furthermore, 160 μl per 500 μg of peptides of 1% NH_4_OH were added and mixed for 5 min. Subsequently, 5% formic acid (160 μl per 500 μg of peptides) was added and mixed. The solution was placed at 4 °C for at least 1 h. The light and heavy dimethyl labelling peptides were combined in a 1:1 ratio and desalted using a Sep-Pak C_18_ column (Waters).

Mixed peptides were subsequently fractionated by using a C_18_-ST column (2.0 mm×50 mm, 5 μm particle size) (TechMate) on the Agilent 1260 system (Agilent Technologies). An elution gradient of 60 min was used for peptide separation with 20 mM ammonium formate in H_2_O (adjusted to pH 10 by 25% NH_3_·H_2_O) as solvent A and 20 mM ammonium formate in 80% acetonitrile (ACN; adjust pH to 10 by 25% NH_3_·H_2_O) as solvent B. The gradient elution profile was composed of 5–25% B for 20 min, 25–45% B for 15 min, 45–90% B for 1 min, then maintained at 90% B for 4 min, followed by 10–95% A for 1 min, and ending with 95% A for 14 min. The flow rate was 0.2 ml min^–1^. UV absorbance was monitored at 216 nm. A total of 60 fractions of 0.2 ml were collected, then concatenated and mixed to obtain 20 fractions. Fractions were dried via speed-vacuum and desalted by the StageTip C_18_ method.

Reverse phase liquid chromatography-electrospray ionization-tandem MS (RPLC-ESI-MS/MS) was used to detect the sample. LC-MS/MS detection was carried out on a hybrid quadrupole-time of flight (TOF) LC/MS/MS mass spectrometer (TripleTOF 5600^+^, AB Sciex) equipped with a nanospray source. Peptides were first loaded onto a C_18_ trap column (5 µm, 5 mm×0.3 mm, Agilent Technologies) and then eluted into a C_18_ analytical column (75 μm×150 mm, 3 μm particle size, 100 Å pore size, Eksigent). Mobile phase A (3% DMSO, 97% H_2_O, 0.1% formic acid) and mobile phase B (3% DMSO, 97% ACN, 0.1% formic acid) were used to establish a 100 min gradient, which consisted of 0 min of 5% B, 65 min of 5–23% B, 20 min of 23–52% B, 1 min of 52–80% B, and the gradient was maintained in 80% B for 4 min, followed by 0.1 min of 80–85% B, and a final step in 5% B for 10 min. A constant flow rate was set at 300 nl min^–1^. MS scans were conducted from 350 amu to 1500 amu, with a 250 ms time span. For MS/MS analysis, each scan cycle consisted of one full-scan mass spectrum (with *m/z* ranging from 350 to 1500 and charge states from 2 to 5) followed by 40 MS/MS events. The threshold count was set to 120 to activate MS/MS accumulation, and former target ion exclusion was set for 18 s.

### Library construction and mass spectrometry database searching

An AS junction library (576 570 entries) was constructed as described previously (Sheynkman *et al.*, 2013; Castellana *et al.*, 2014; Walley and Briggs, 2015) with minor modifications. In brief, six frame translations, comprising three frames on the forward strand and three frames on the reverse complement strand, were used to construct the AS junction library. Additionally, a frame library was constructed using all transcripts annotated in the reference annotation file by six frames. The redundant sequences were then removed from translated sequences at the first step. Peptide sequences >6 amino acids were attached to the UniProt rice japonica database for subsequent database search. Raw spectrum data generated from both qualitative and quantitative proteomics were searched with the ProteinPilot software (v5.0, AB SCIEX) using pre-set parameters. All data were filtered at a 1% FDR with at least one peptide at the 95% confidence level calculated automatically by the ProteinPilot software (Zhu *et al.*, 2017). For quantitative proteomics, data were searched against UniProt and self-constructed databases using the following parameters: sample type, dimethyl (0, +4) quantitation; cys alkylation, iodoacetamide, digestion, trypsin. The search effort was set to rapid ID. For differentially expressed protein (DEP) analysis, proteins with a fold change of >1.2 or <0.8 (*P*-value <0.05) are considered as DEPs in this study.

### Quantitative real-time PCR validation of AS transcripts

Total RNA (~5 μg) was reverse-transcribed into cDNA by using the Superscript First-Strand Synthesis System (Invitrogen, USA) following the manufacturer’s instructions. Quantitative real-time PCR (qRT-PCR) was conducted as described previously (Zhu *et al.*, 2013) and based on previous standard rules established in the plant research area (Udvardi *et al.*, 2008). Two independent experiments, with three replicates of each experiment, were performed for each gene, and *OsACTIN1* was used as an internal reference gene. The resulting products of qRT-PCR were subjected to DNA sequence analysis. Isoform-specific primers used for alternatively spliced isoform identification are listed in Supplementary Table S9.

### Data submission

The rice transcriptome data have been uploaded to the Sequence Read Archive (https://www.ncbi.nlm.nih.gov/sra) under Bioproject PRJNA451248. The raw data of qualitative and quantitative proteomics have been submitted to the PRIDE PRoteomics IDEntifications (PRIDE) database with accession number PXD010923.

## Results

### Improvement of the analytical pipeline and experimental conditions

The analytical pipeline used in this study is presented in Supplementary Fig. S1. Improvements have been made since the last bioinformatic flowchart (Zhu *et al.*, 2017). The identification and quantification procedures of AS events were simplified for subsequent GO and KEGG analyses. In addition, refinement of redundancy and error check steps further improved the accuracy of identification. In this study, AS events such as an AFE (alternative first exon) and ALE (alternative last exon) caused purely by an alternative transcription start and polyadenylation have been removed to differentiate AS modification further from other transcriptional or post-transcriptional mechanisms. To distinguish 5' donor sites and 3' acceptor sites, we further divided AE (alternative exon) events into AE5' and AE3' for further bioinformatic analysis. Furthermore, incorporation of quantitative proteomics yielded more information on steady-state protein levels in comparison with qualitative proteomic profiling, which can only identify the presence of translated peptides (Zhu *et al.*, 2017). For testing samples, we chose dry seeds of japonica rice (Nipponbare) treated with hypoxia (3% O_2_) for 6 h in comparison with air controls under complete darkness. This treatment will help us to understand the short-term responses at both transcript and protein levels during hypoxia when seeds start to germinate. Large amounts of samples were harvested for the following three profiling experiments: short-read RNA-seq, and qualitative and quantitative proteomics. Prior to these experiments, we tested conditions for hypoxia treatment by phenotypic and biochemical evaluations to ensure the severity of stress treatment conditions (Supplementary Figs S2, S3). In addition, we have compared 49 up-regulated anaerobic marker genes highlighted in previous publications with our data set (Lasanthi-Kudahettige *et al.*, 2007; Narsai *et al.*, 2009, 2011, 2015; Sadiq *et al.*, 2011; Hsu and Tung, 2017). Among 21 genes detected in this study, 19 genes showed consistency in their differential regulation, but at a lower magnitude (Supplementary Fig. S4A). We used qRT-PCR to validate this expression further. In total, 18 of 19 genes showed a similar expression pattern as the result of our RNA-seq data (Supplementary Fig. S4B), indicating the efficacy of hypoxic treatment using 3% O_2_ in our system.

### Completely different sets of genes undergo AS in response to hypoxia during rice seed germination

**Fig. 1.**
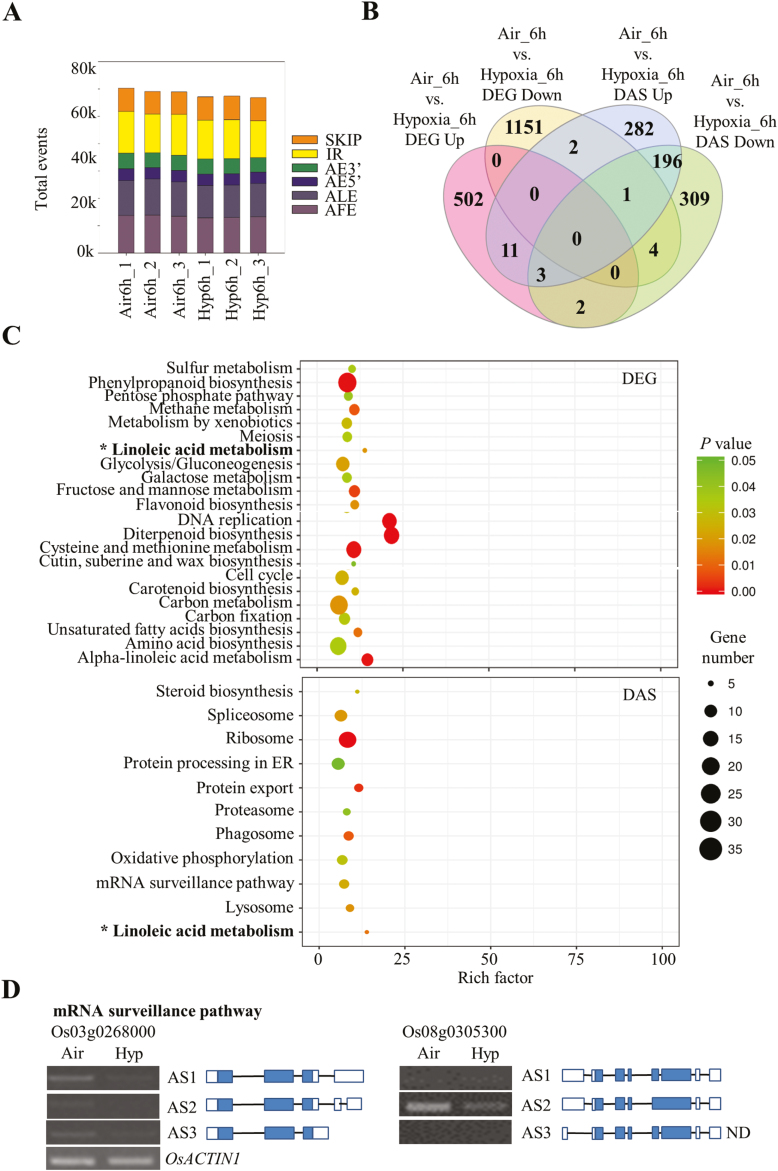
Identification and comparison between the data sets of differentially expressed genes (DEGs) and differentially expressed alternative splicing (DAS) events during rice hypoxic germination. (A) Statistics of the identified alternative splicing (AS) events and types. ALE, alternative last exon; AFE, alternative first exon; SKIP, exon skipping; IR, intron retention; AE5', alternative donor; AE3', alternative acceptor. (B) The Venn diagram represents unique and shared genes between DEG and DAS data sets. (C) Gene Ontology enrichment analysis between DEG and DAS data sets. (D) RT-PCR validation of the DAS events in the mRNA surveillance pathway. Air, air control; Hyp, hypoxia; ND, not detected. Gene models of each isoform are indicated (blue, coding region; white, non-coding UTRs; not to scale).

**Fig. 2.**
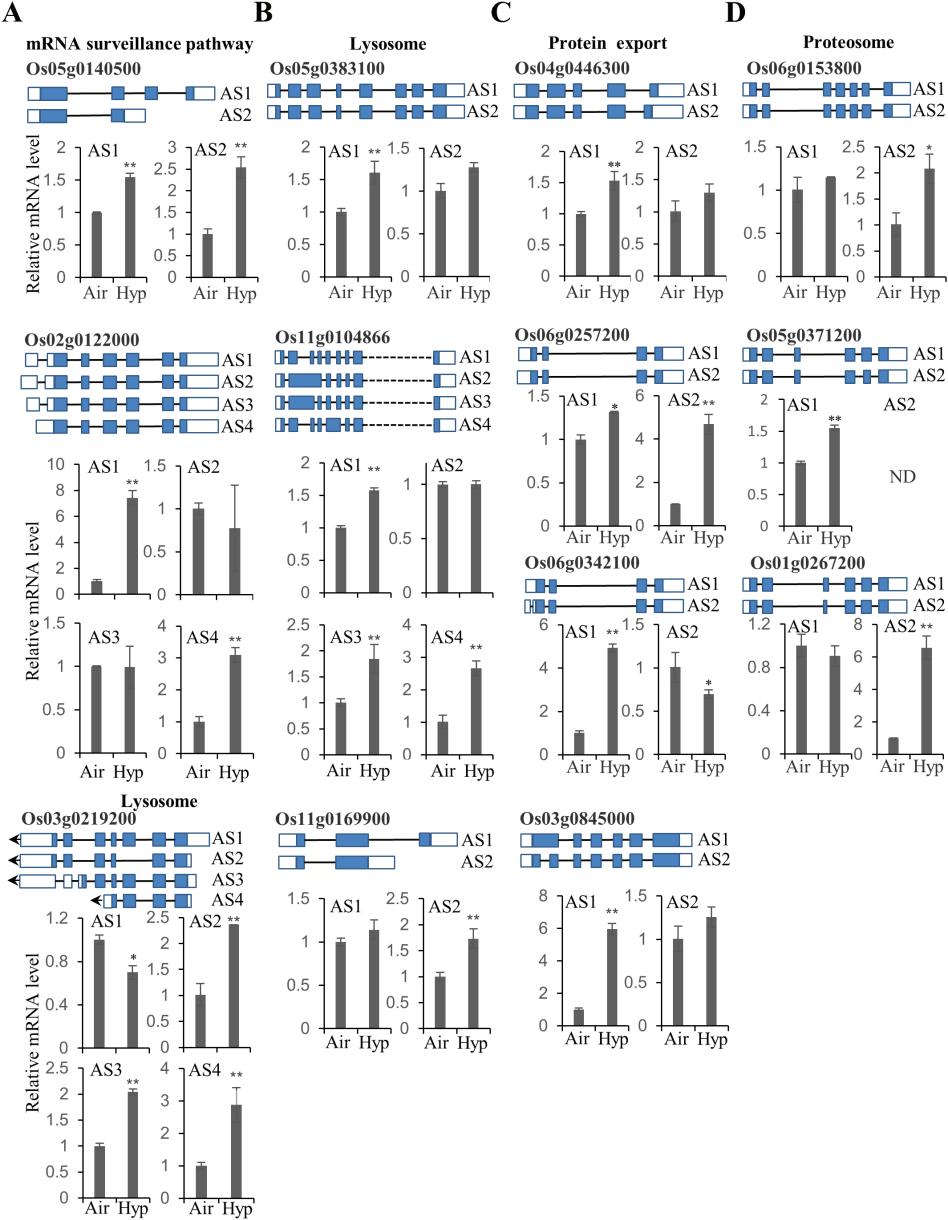
qRT-PCR validations of differentially expressed AS (DAS) events. Validation of DAS events detected in KEGG enrichment analysis. DAS events involved in categories of (A) mRNA surveillance pathway and lysosome, (B) lysosome, (C) protein export,and (D) proteasome were verified by qRT-PCR analysis from three biological replicates. *OsACTIN1* was used as an internal reference gene. Mean values ±SD are presented (*n*=3). ** and * represent that the mean values of the hypoxia-treated (Hyp) group are significantly higher or lower than those in the air control (Air), at *P*<0.01 and *P*<0.05, respectively. Gene models of each isoform are indicated (blue, coding region; white, non-coding untranslated regions; not to scale).

Approximately 1.32 billion raw reads in total averaging 200 million reads per sample were obtained from RNA-seq (Supplementary Table S1). Among these, 1.25 billion clean reads were subjected to the mapping process. On average, ~95% were uniquely mapped to the genome and used for subsequent bioinformatic analysis (Supplementary Table S1). For AS identification, each sample identified >75 000 AS events. In total, 10 253/26 848 (38.2%) annotated intron-containing genes were observed to exist as AS events in rice seeds. Approximately 6.4% (1729/26 848) more intron-containing genes were observed in comparison with the original annotation file. Slightly different from a previous AS analysis in ABA-treated Arabidopsis seedlings (Zhu *et al.*, 2017), AFEs, ALEs, and intron retention (IR) remained as the three most abundant AS events in all the samples (Fig. 1A). Among these three AS event types, AFEs and ALEs caused variable 5'- and 3'-untranslated ends, which may affect the efficiency of translation or the stability of corresponding transcripts (Andreassi and Riccio, 2009; Sonmez *et al.*, 2011; Jenal *et al.*, 2012). For example, hidden sORFs from the 5' end of transcripts encoding short peptides have the ability to regulate translational efficiency of target transcripts (Laing *et al.*, 2015), whereas polyadenylation at the 3' end of transcripts is well known to affect the localization and stability of the transcripts (de Lorenzo *et al.*, 2017). When the data set of DEGs (Supplementary Table S2) was compared with the DAS data set (Supplementary Table S3), >95% were not the same (Fig. 1B). Only 23 genes were differentially regulated at both transcription and post-transcriptional levels (Fig. 1B). This suggests that AS may play an important and distinctive role during rice hypoxic germination. Subsequent GO enrichment analysis also confirmed the result from the Venn diagram (Fig. 1B; Supplementary Fig. S5). In several cases, DEGs and DASs did not co-exist in the same secondary GO category (Supplementary Fig. S5). Fourteen isoforms of seven genes in the DAS data set were assembled and validated by qRT-PCR. In total, six of these genes were consistent with the data from RNA-seq analysis, suggesting the reliability of AS identification and quantification from the analytical pipeline (Supplementary Fig. S6). With the exception of categories related to linoleic acid metabolism, the majority of DEGs and DASs were not enriched in the same KEGG category (Fig. 1C), suggesting that the DAS category is a different group of genes in response to hypoxic germination. The majority of pathways enriched in the DEG data set were closely related to cellular metabolism (e.g. pentose phosphate pathway, glycolysis/gluconeogenesis, fructose and mannose metabolism, etc.) and cell growth (meiosis, DNA replication, and the cell cycle, etc.). Whereas some regulatory pathways were specifically over-represented in the DAS data set, such as spliceosome, ribosome, endoplasmic reticulum (ER) protein processing, protein export, proteasome, phagosome, oxidative phosphorylation, and mRNA surveillance pathway, implying that these pathways may play an essential role in AS-mediated responses under rice hypoxic germination. Gene members in several pathways have been selected for RT–PCR and qRT-PCR validation (Figs 1D, 2). Some splicing isoforms of corresponding genes showed differential expression under hypoxic treatment, indicating their potential role in response to rice hypoxic germination.

### Qualitative proteomic identification reveals that hypoxia-regulated AS events are likely to be translated

**Fig. 3.**
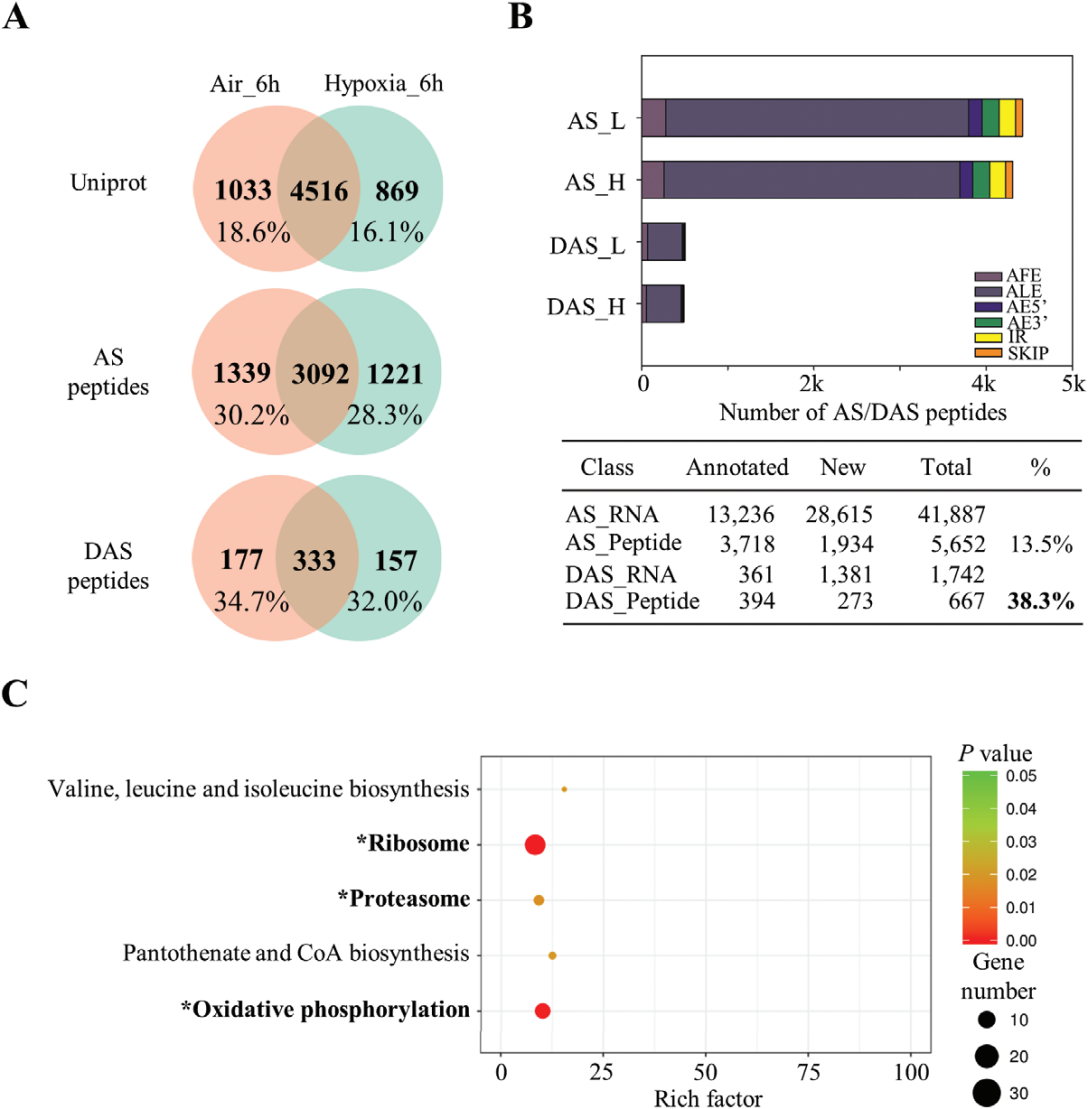
Qualitative proteomic identification of alternatively spliced peptides. (A) Venn diagram representation of qualitative proteomic identification using UniProt, AS, and DAS databases in air control and hypoxia-treated samples. (B) Identification of alternatively spliced peptides and classification (upper panel). L, air control; H, hypoxia treatment; ALE, alternative last exon; AFE, alternative first exon; SKIP, exon skipping; IR, intron retention; AE5', alternative donor; AE3', alternative acceptor. Summary of identified AS/DAS events and peptides (lower panel). (C) KEGG pathway enrichment analysis of DAS peptides in qualitative proteomics. Pathways marked with an asterisk are repeatedly found in both transcriptome and qualitative proteomic data sets.

To characterize further the translational products of identified AS events, we carried out a qualitative proteomic profiling using MS/MS for both control and hypoxia-treated samples (Alfaro *et al.*, 2014; Tavares *et al.*, 2015; Zhu *et al.*, 2017). Proteomic analysis this time generated 547 545 and 485 392 high-quality spectra for control and hypoxia-treated samples, respectively. Approximately 5549 and 5385 proteins were identified using the UniProt database (Fig. 3A). Among these, 18.6% and 16.1% of identified proteins were uniquely present in control or hypoxia-treated samples, respectively, serving as good candidates for further functional characterization. A subsequent AS junction library search identified 4431/4313 peptides from AS events (41 887) and 510/490 peptides from DAS events (1742) for control/hypoxia-treated samples, respectively (Fig. 3A, B). Among these, ~70% of peptides were shared by both samples. Intriguingly, far fewer AFE events could be detected at the peptide level in comparison with ALE events (Fig. 2B). Furthermore, 13.5% of the total AS events (5652/41 887) were translated into peptides, suggesting that the majority of AS transcripts may be degraded by RNA surveillance mechanisms such as nonsense-mediated mRNA decay (NMD) (Nicholson *et al.*, 2010; Drechsel *et al.*, 2013). In contrast, an elevated percentage (38.3%) of DAS events could be translated into peptides in all alternatively spliced types (Fig. 3B), indicating their potential role in response to hypoxic stress during rice germination. Similar observations have been reported in abscisic acid (ABA)-treated Arabidopsis seedlings (Zhu *et al.*, 2017). The higher percentage of translation detected in hypoxia-treated samples indicates that thousands of alternatively spliced proteins are translated under hypoxic conditions during rice germination. Furthermore, most of these alternatively spliced peptide-encoding genes were not present in the DEG list analysed by a conventional RNA-seq pipeline.

In addition, ~68.3% of AS events identified in this study were not annotated in the genome and thus were marked as new features for rice genome annotation (Fig. 3B). Also, 40.9% of the DAS peptides were not present in the current version of the annotation, which suggests the translation of new protein isoforms during rice germination in response to hypoxia. DAS peptides were subjected to KEGG enrichment analysis (Fig. 3C). For example, some KEGG terms including, ribosome, proteasome, and oxidative phosphorylation, were repeatedly enriched in both RNA-seq and qualitative proteomic data sets, giving protein evidence of these splicing isoforms in response to hypoxia treatment.

### Quantitative proteomics indicates that the expression of protein and transcripts is correlated at the AS level

**Fig. 4.**
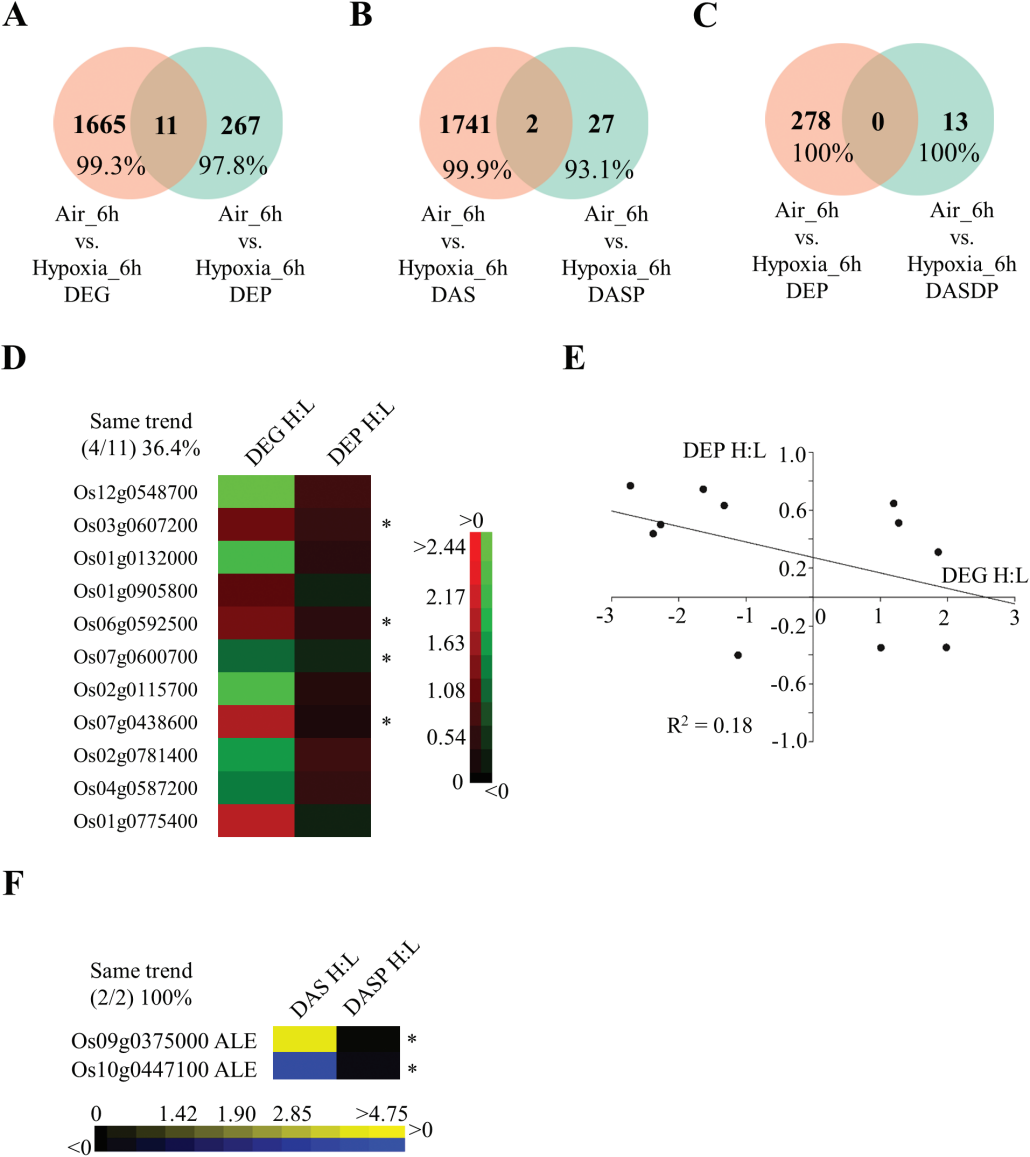
Comparison between proteomic and transcriptomic data sets. Venn diagram representation of (A) differentially expressed genes (DEGs) versus differentially expressed proteins (DEPs), (B) differentially expressed AS (DAS) events versus differentially expressed AS peptides (DASDPs), (C) DEPs versus DASDPs. Heatmap representation (D) and correlation analysis (E) of overlapping genes between DEGs and DEPs. (F) Heatmap representation of overlapping genes between DASs and DASDPs. ALE, alternative last exon; an asterisk represents the regulation of transcripts and proteins with the same trend in corresponding data sets; H:L, hypoxia versus air control.

To find the relationship between the protein abundance and corresponding transcripts at the AS level, quantitative proteomics were conducted using the dimethyl labelling method. In total, 10 946 proteins were identified from this approach and 4566 of them were quantified Supplementary Table S4). Among these, 278 DEPs and 29 peptide evidence of DAS events (DASPs) were identified by quantitative and qualitative proteomics, respectively (Fig. 4A, B). Thirteen DASPs identified by qualitative proteomics were further found to be differentially expressed in quantitative proteomics and referred to as as DASDPs. Amongst these, none of them was shared with the DEP data set (Fig. 4C), including abundant seed storage proteins (Supplementary Table S5). Similar to previous parallel analyses (Bai *et al.*, 2015; Marmiroli *et al.*, 2015), much less overlap was observed between DEPs and DEGs, DASs and DASPs, as well as DEPs and DASDPs (Fig. 4A–C). Construction of a customized protein library leads to identification and quantification of novel proteins during rice germination. Only 11 genes were identified as both DEGs and DEPs with low correlation (*R*^2^=0.18) of their expression levels (Fig. 4D, E), suggesting the existence of post-transcriptional regulation for most of the transcripts. Although two genes were detected in both the DAS and DASP data sets, the expression of their transcripts and proteins showed the same trend (Fig. 4F), indicating that quantification at the alternatively spliced isoform level may provide more accurate data representation for both transcripts and proteins than the conventional quantification method used in RNA-seq and proteomics. However, more data are required to confirm this hypothesis. In addition to the effect of post-transcription, the low overlap of DEP/DASDP with DEG/DAS data sets may be explained by the relatively low throughput and coverage of the MS-based proteomic method.

### Construction of a customized protein library leads to novel protein identification and quantification during rice hypoxic germination

**Fig. 5.**
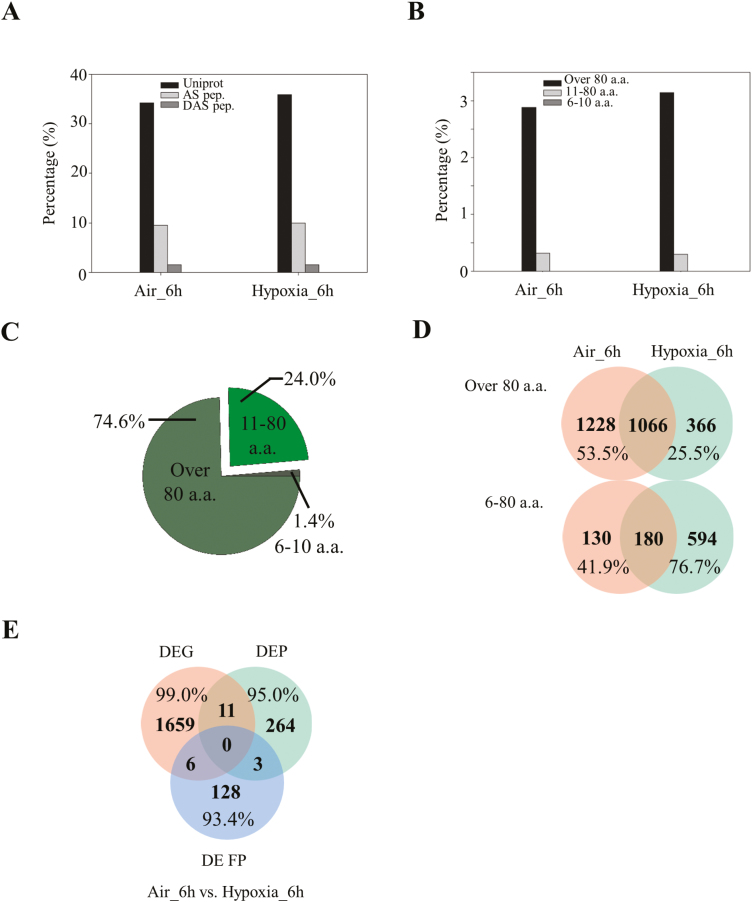
Novel protein/peptide identification. (A) Summary of the spectrum usage of the data from qualitative proteomics using UniProt, AS, and DAS databases. (B) Spectrum usage of the data from qualitative proteomic using the six frame translated protein database. (C) The pie chart represents the percentage distribution of identified novel proteins/peptides. (D) Venn diagram representation of identified novel proteins/peptides in control and hypoxia-treated samples. (E) The Venn diagram represents the shared and unique genes among differentially expressed genes (DEGs), differentially expressed proteins (DEPs), and differentially expressed frame proteins (DE FPs).

Similar to previous findings (Zhu *et al.*, 2017), the spectra usage for protein identification was ~40–50% in this study (Fig. 5A) using both UniProt and AS junction libraries as input files. An increasing number of publications suggest that single transcripts are able to be translated into multiple proteins by using ATI sites (Brar and Weissman, 2015). This indicates that a large number of novel proteins or short peptides are yet to be identified, and this is caused by incomplete genome annotation (Kim *et al.*, 2014). Thus, a six frame translation library was constructed using the combination of assembled cufflink files during RNA-seq analysis and reference annotation files based on previously published methods (Castellana *et al.*, 2008; Zhu *et al.*, 2017). The database searching identified thousands of novel proteins and peptides translated by a different frame from the same transcripts, with 74.6% of proteins longer than 80 amino acids, 24.0% of proteins/peptides from 11 to 80 amino acids, and 1.4% of peptides from 6 to 11 amino acids (Fig. 5B, C). Among these, 2294/1432 novel proteins (>80 amino acids) and 310/774 novel proteins or peptides (6–80 amino acids) were identified in control/hypoxia-treated samples, respectively (Fig. 5D). This observation provides further evidence of additional coding ability for proteins and short peptides by using ATI sites. Additionally, an increasing number of short peptides (774) were detected in hypoxia-treated samples in comparison with air controls (310), suggesting that short peptides may play an important role in response to hypoxia during rice germination. Intriguingly, 137 novel proteins were quantified at a second frame of known transcripts. Few of these overlapped with DEG and DEP data sets, indicating that most of these proteins can only be detected by proteomic analysis using the customized library. This set of genes served as a source of novel candidates for further investigation of hypoxic responses during rice germination.

### The conventional 5'-splicing sites are less conserved in rice seeds under normal conditions and under hypoxia treatment

**Fig. 6.**
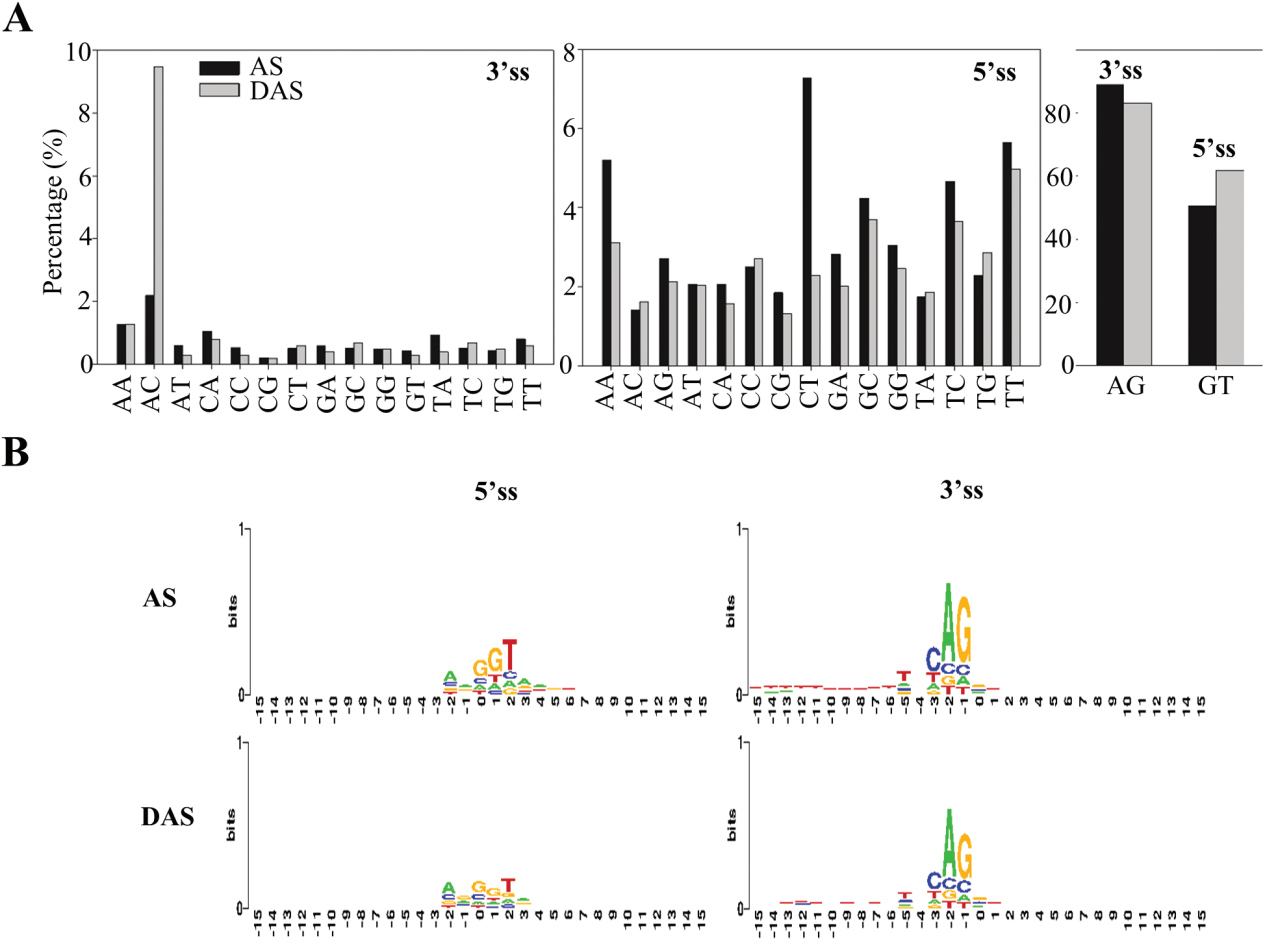
Splicing site (ss) recognition under hypoxia stress. (A) Statistical analysis of the ss between the total AS events and the hypoxia-affected DAS events. (B) Conservation analysis using sequences located at exon–intron junctions.

To investigate further the splicing characteristics between total AS and hypoxia-affected DAS data sets, statistical analysis of splicing site conservation was performed. Conventionally, U2-type splicing sites (5'-GT–AG-3') are conserved and account for 90% of total splicing sites among plant species (Will and Luhrmann, 2011). In this study, the 3'-splicing site (AG) was relatively conserved and accounted for >80% in both control and hypoxia-treated samples (Fig. 6A). An extra ‘C’ was identified as a conserved sequence in both AS and DAS data sets (Fig. 6B). Thus, 3'-splicing sites were identified as ‘CAG’ in rice seeds, and the hypoxia treatment did not change this signature (Fig. 6B). However, there was a decrease in the ‘AG’ proportion in hypoxia-treated samples, which was associated with the increase in the proportions of several other splicing site sequences, especially ‘AC’. In contrast, the conventional 5'-splicing site (GT) accounted for only 50% of total AS and was increased to ~60% in the hypoxia-affected DAS data set (Fig. 6A). Meanwhile, non-conventional 5'-splicing sites such as ‘AA’ and ‘CT’ were greatly reduced in the DAS data set in comparison with the AS data set, suggesting its role in response to hypoxia stress (Fig. 6A). In addition, similar results were obtained by conservation analysis; the ‘GGT’ signature was obtained in both AS and DAS data sets (Fig. 6B). Further investigation of splicing sites among alternatively spliced types demonstrated that AFEs were responsible for the ‘GT’ reduction in both AS and DAS data sets (Supplementary Fig. S7). Although 3'-splicing sites were more conserved, certain types of non-conventional splicing sites were induced among the specific alternatively spliced types in the DAS data set in comparison with the AS data set, such as 3'-TG and 3'-TT in AE5', 3'-AC and 3'-GG in AE3', 3'-GC and 3'-TG in IR, and 3'-GC in exon skipping (Supplementary Fig. S7). This result indicates that AS regulation under hypoxia stress may be caused by alternative recognition of the sequence of splicing sites.

### Splicing factors are enriched in differentially expressed AS events

**Fig. 7.**
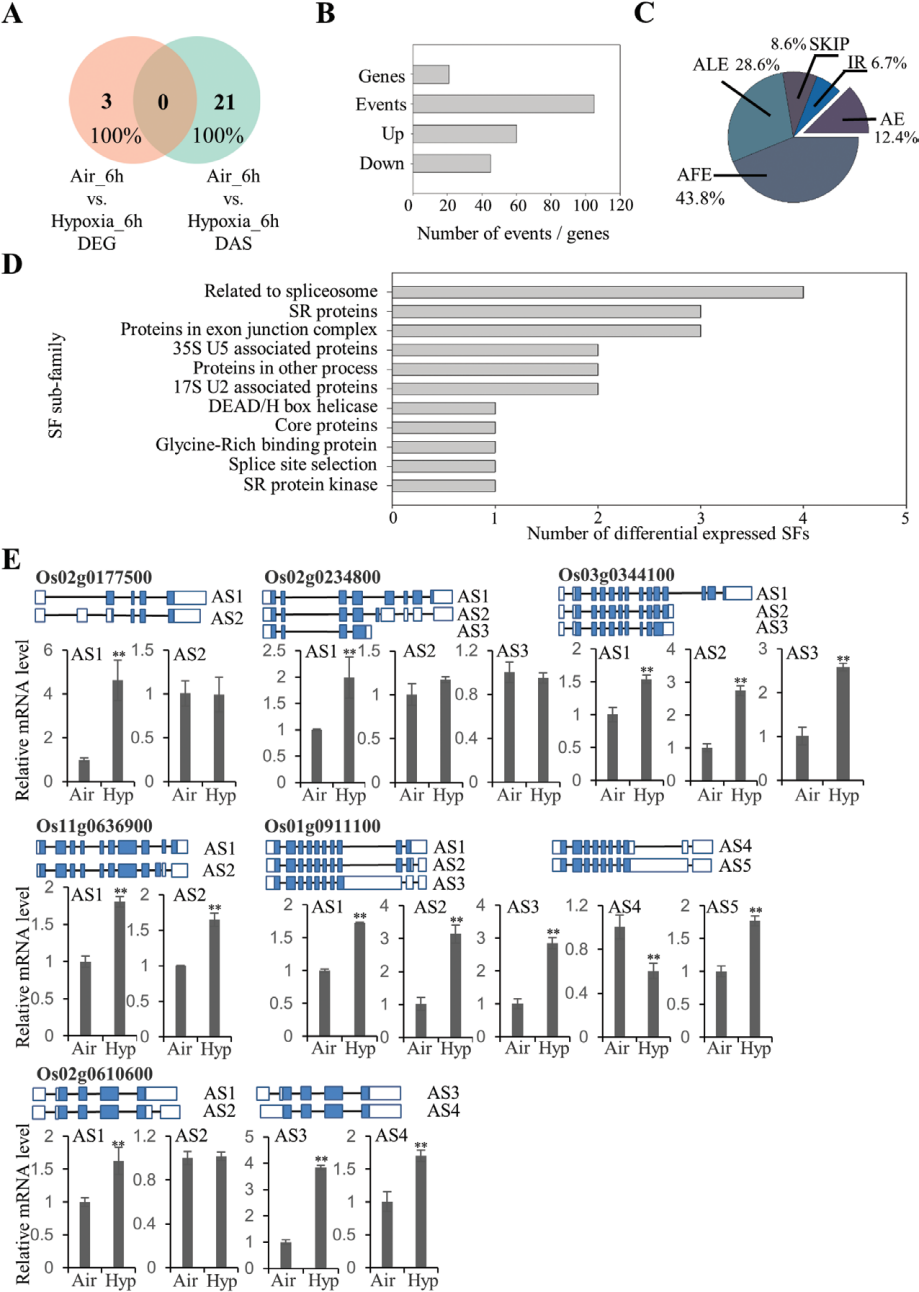
Splicing factors involved in hypoxia responses during rice germination. (A) The Venn diagram represents identified splicing factors between DEG and DAS data sets. (B) Statistics of hypoxia-affected DAS genes and events of splicing factors. (C) Pie chart distribution of the DAS events belonging to splicing factors. (D) Subgroup classification of splicing factors identified in DAS events. (E) qRT-PCR validation of DAS events detected in the spliceosome. DAS events in the category of spliceosome were verified by qRT-PCR analysis from three biological replicates. *OsACTIN1* was used as an internal reference gene. Mean values ±SD are presented (*n*=3). ** and * represent that the mean values of the hypoxia-treated (Hyp) group are significantly higher or lower than those in the air control (Air), at *P*<0.01 and *P*<0.05, respectively. Gene models of each isoform are indicated (blue, coding region; white, non-coding UTRs; not to scale).

To understand further the underlying mechanism of AS regulation under hypoxia stress, splicing factors in rice were subjected to further analysis. Three genes were found to be differentially expressed in the DEG data set (Fig. 7A), suggesting that they may not respond to transcriptional regulation. In contrast, a total of 105 AS events from 21 splicing factor-related proteins were observed in the DAS data set (Fig. 7A, B), and none of them was found in the DEG data set, indicating that these splicing factors are specifically regulated by post-transcriptional mechanisms. Among these, 60 AS events were up-regulated, whereas 45 AS events were down-regulated (Fig. 7B). In detail, 43.8% of AS events were AFEs and ALEs, accounting for 28.6% (Fig. 7C). The remaining three alternatively spliced types accounted for 27.7% of the total AS events (Fig. 7C). According to the classification in the splicing-related gene database (SRGD; http://www.plantgdb.org/SRGD/index.php), the 21 genes observed in the DAS data set were classified into 11 subgroups (Fig. 7D) from core splicing components to auxiliary factors. In addition those splicing factors enriched in the KEGG term of the spliceosome (Fig. 1C) were chosen for qRT-PCR validation (Fig. 7E). Some isoforms of selected splicing factors were differentially expressed under hypoxia treatment, suggesting that the change of AS in splicing components may be crucial in response to hypoxia stress during rice germination.

## Discussion

### The discovery of a hidden network of AS in response to hypoxic stress during rice germination provides additional targets in the study of hypoxia

AS produces multiple RNA isoforms for each locus. Each isoform may encode one protein isoform as well, which greatly expands the genome coding ability. Additionally, the discovery of two new alternatively spliced types, AFEs and ALEs, has revealed the great potential to generate alternatively spliced isoforms (Yan and Marr, 2005; de Klerk and ’t Hoen, 2015; Zhu *et al.*, 2017). In this study, ~97.2% (787/810) of the DAS genes had no differences at the gene expression level, suggesting that AS control of transcripts is completely separated from the conventional DEG group (Fig. 1B). Moreover, increasing evidence reveals that protein isoforms generated by alternatively spliced transcripts have the ability to alter protein subcellular localization, protein–protein interaction networks, and protein stability due to the presence or absence of certain motifs (Buljan *et al.*, 2012; Ellis *et al.*, 2012). Here, a total of 18 protein isoforms from eight genes were predicted to have a different subcellular localization (Figs 1D, 2, 7E; Supplementary Fig. S6; Supplementary Table S6). In addition, the 667 alternatively spliced peptides identified in both control and hypoxia-treated samples provided protein evidence of alternatively spliced transcripts and may serve as good candidates for further functional characterization (Fig. 3A, B). These genes were distributed in a variety of biological pathways, including amino acid biosynthesis, ribosome and proteasome pathway, pantothenate and CoA biosynthesis, and oxidative phosphorylation, and were not selected for further investigation by the first round of screening using DEGs as criteria amongst large-scale transcriptome analysis, suggesting that AS responses are embedded in various biochemical processes under hypoxia stress.

Transcriptomic studies have shown that low oxygen induces myriad gene responsiveness in terms of transcript abundance (Lasanthi-Kudahettige *et al.*, 2007; Narsai *et al.*, 2009, 2011, 2015; Sadiq *et al.*, 2011; Hsu and Tung, 2017). Accordingly, transcriptional regulation in oxygen-sensing pathways has been extensively studied in plants. Key regulators, such as ERFVII transcription factors, have been substantially characterized (Fukao *et al.*, 2009; Hattori *et al.*, 2009; Hinz *et al.*, 2010; Licausi *et al.*, 2010). However, few studies have been carried out to unravel AS regulation under hypoxia. In the current study, AS analysis indicates that the conventional splicing sites are not conserved at the 5' position in rice seeds (Fig. 6A, B; Supplementary Fig. S7). Major regulators defined as splicing factors within the assembled spliceosome have been characterized as participating in AS site determination (Golovkin and Reddy, 1996; Kalyna *et al.*, 2006; Pomeranz Krummel *et al.*, 2009; Will and Luhrmann, 2011; Kondo *et al.*, 2015; Yoshida *et al.*, 2015). Although several splicing factors have been reported to be involved in stress responses (Rühl *et al*., 2012; Feng *et al.*, 2015), none of them is related to hypoxia responses. In our results, a 7-fold increase in the number of splicing factors (21 in DASs; 3 in DEGs) was found in comparison with the DEG data set (Fig. 7), suggesting the importance of those proteins in splicing site recognition. Over 100 AS events in these 21 splicing factors were affected during hypoxia treatment, which may greatly alter the protein isoforms of these proteins in comparison with the control group. Subsequently, hypoxia may change the composition and conformation of spliceosomes by recruiting different protein isoforms of splicing factors, which may in turn lead to a different choice of splicing site sequence recognition. This may explain the increment of the proportion of certain non-conventional splicing sites during rice hypoxic germination (Supplementary Fig. S7). Furthermore, plants prioritize translation of selected transcripts during hypoxia, as part of an energy-conserving strategy (de Lorenzo *et al.*, 2017; Zhao *et al.*, 2017). Here, the integration of qualitative proteomic data implies that hypoxia-responsive AS events are more likely to be translated in comparison with non-responsive events (Fig. 3B, lower panel), providing protein evidence for the potential role of these alternatively spliced isoforms in response to hypoxia stress. Therefore, our results suggest that AS is an independent pathway from transcriptional repression in response to hypoxia during rice germination. The majority of members in this pathway remain to be elucidated.

### Alternative cellular pathways are activated by AS under hypoxia treatment

Several pathways were found to be over-represented under AS-mediated responses during rice hypoxic germination. mRNA surveillance, such as NMD, has long been demonstrated to play an important role in controlling mRNA stability and abundance before translation (Nicholson *et al.*, 2010; Drechsel *et al.*, 2013). It has been reported that NMD is closely related to the exon junction complex (EJC) of the splicing machinery in both animals and plants (Shaul, 2015). In Arabidopsis, hypoxia-responsive ERFs, HRE1 and HRE2, have been proposed to be regulated by post-transcriptional mechanisms for their mRNA stability (Licausi *et al.*, 2010). From our data set, isoforms of several components belonging to the EJC complex (e.g. Os08g0305300, *OsSMG7* and Os05g0140500, *OsY14a*) were observed to be differentially regulated (Nyikó *et al.*, 2013), indicating their potential function in surveillance of newly spliced RNA isoforms under hypoxia. Evidence shows that the status of the spliceosome will be affected under hypoxia in animal tissues (Schmidt-Kastner *et al.*, 2008). Splicing factors such as serine-arginine (SR) proteins are activated under hypoxic conditions by phosphorylation (Jakubauskiene *et al.*, 2015). However, the responsiveness of the spliceosome under hypoxia treatment remains to be elucidated *in planta*. In this study, a variety of splicing components have been identified to show differential expression under hypoxia treatment. Among these, six isoforms from two SR proteins (Os03g0344100, *SR32* and Os02g0610600, *RSZ23*) were induced by hypoxia treatment (Fig. 7E). Although multiple isoforms of SR proteins have been detected in different rice tissues (Peng *et al.*, 2013), no evidence has linked them to hypoxia stress responsiveness before. Here, we hypothesize that changes in splicing factors under hypoxia are crucial for downstream AS regulation. However, less information can be found on these identified splicing factors from current databases. Further functional characterization is required to confirm their roles in response to hypoxia. Besides post-transcriptional regulatory pathways, processes related to protein export, lysosomes, and proteasomes were observed to play a role during hypoxic germination (Figs 1, 2). The enhancement of some splicing isoforms in the protein export process (Fig. 2C) may effectively help plants to survive during hypoxia conditions. Furthermore, the lysosome is a place where cells recycle building materials or undergo detoxification (Chen *et al.*, 2015). A recent study shows that hypoxia may rapidly induce autophagy, which is a highly conserved mechanism in eukaryotes to target cellular components to the lysosome for recycling purposes (Chen *et al.*, 2015). Thus, the newly formed isoforms of lysosomal genes may be responsible for the survival under hypoxia stress. Similarly, protein degradation has been considered as a major mechanism in response to hypoxia in both animals and plants (Huang *et al.*, 1998; Gibbs *et al.*, 2011; Licausi *et al.*, 2011). Significant misfolded proteins generated under hypoxia need to be degraded in order to maintain cellular function. New isoforms formed in this process may have superior efficacy to degrade misfolded proteins, thus alleviating the stress conditions resulting from hypoxia treatment. Intriguingly, transcriptional regulation focused on the control of cellular metabolic levels and growth factors, whereas AS aims to produce new protein isoforms that are mainly involved in degradation, post-transcriptional regulation, and transport processes. These two complementary mechanisms may facilitate rice seed survival under hypoxia during germination.

### Thousands of novel proteins or peptides resulting from alternative translation participate in the hypoxia response during rice germination

In addition to the protein diversity resulting from AS, proteins encoded from a second frame of the same transcript or from annotated non-coding regions also contribute to genome coding ability (Jensen *et al.*, 2013; Wade and Grainger, 2014). Specifically, a considerable number of unannotated proteins were detected using a customized library by six frame translation (i.e. three in the forward strand and three in the reverse complement strand). The coding ability of one transcript using a second frame has been widely studied in animals but is rarely reported in plants (de Klerk and ’t Hoen, 2015). One example from plant systems is an α-enolase gene (*LOS2*) in Arabidopsis that encodes a cmyc-binding protein (MBP)-like protein by alternative translation. This MBP-like protein affects ABA responses, and its protein level is regulated by the E3 ligase SAP5 (Kang *et al.*, 2013). Furthermore, the existence of uORFs in the 5'-untranslated regions (UTRs) of certain transcripts may lead to a feedback regulation of translation efficiency (Laing *et al.*, 2015). From our results, a total of 2660 putative proteins of >80 amino acids and 904 proteins/peptides ranging from 6 to 80 amino acids have been identified (Fig. 5D). A total of 960 of these proteins/peptides were specifically induced under hypoxia treatment, suggesting that they are new players involved in hypoxia responses. In particular, when we re-mapped identified frame peptides (6–80 amino acids) back to rice transcripts, a total of 58 and 150 peptides were found to be located at the 5'-UTRs of corresponding genes in air control and hypoxia-treated samples, respectively (Supplementary Tables S7, S8). Furthermore, a total of 137 novel proteins were quantified by proteomic analysis, 128 of which were not present in the DEG and DEP lists (Fig. 5E), demonstrating that the usage of a customized library combined with quantitative proteomics is essential for this kind of novel protein/peptide identification.

### Proteogenomic approach evolves as a new-generation method to analyse omics-based data sets

Large profiling methods have been applied in plant research to study various developmental processes or stress responses. However, individual approaches such as transcriptome or proteome analysis are restricted by their defects in experimental conditions and analytical pipelines. For example, pure transcriptome analysis is affected by the corresponding reference genome annotation. Pure proteomic methods are limited by currently available protein libraries, which were generated based on incomplete genome information (Zhu *et al.*, 2017). Thus, proteogenomics, a method incorporating transcriptomic and proteomic data sets, represents a new generation of analytical approaches for deeper understanding of the functional importance of potential genome coding ability (Castellana *et al.*, 2008; Kumar *et al.*, 2016). First, this analytical approach is able to determine which alternatively spliced isoforms will be translated into proteins and thus can differentiate between regulation of mRNA degradation and translational control (Nicholson *et al.*, 2010; Drechsel *et al.*, 2013). Secondly, in combination with quantitative proteomics, proteogenomic analysis links the protein evidence to their transcript changes to give an accurate protein abundance for each transcript isoform during the analysis. Low correlation of the expression levels between proteins and transcripts will be improved when using this type of analytical pipeline (Fig. 4D, E). This in turn will reveal valuable targets that are truly regulated at the transcript and protein levels with the same trend. Finallly, coupled with a self-constructed protein library, this method enhances the identification of novel proteins/peptides (Fig. 5) that are potential hidden regulatory components in plant development or stress responses. However, this approach can be further improved from its current version. For example, using strand-specific library construction in short-read RNA-seq analysis can enhance the accuracy and reduce the redundancy of subsequent protein library construction. Furthermore, using the third generation of sequencing methods, such as single molecule long-read sequencing, can aid in the precise identification of full-length transcripts for accurate identification of AS (Zhu *et al.*, 2017). In addition, increased coverage of proteomic analysis will provide more insight regarding the apparent lack of correlation between transcript and protein levels. The incorporation of SWATH (sequential window acquisition of all theoretical spectra-mass spectrometry)-based quantitative proteomics (Zhu *et al.*, 2016a, b) and two or more enzyme digestion steps may achieve better results than those of the current study.

### Conclusion

**Fig. 8.**
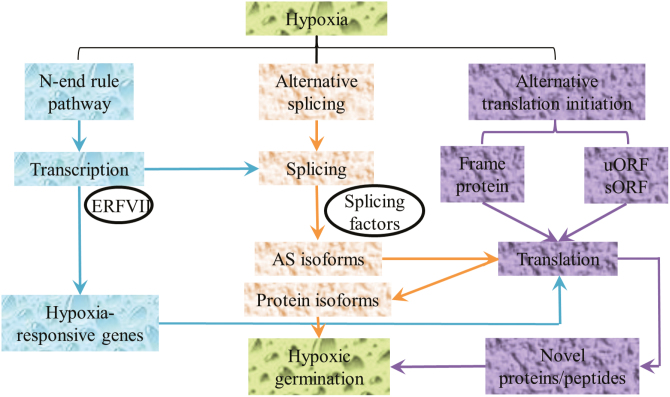
Model of alternative splicing and alternative translation initiation involved in the hypoxic germination pathway. Summary model of the rice genome using its coding ability to produce diverse functional proteins during hypoxic germination. The traditional transcriptional pathway (blue) has been well studied. The parallel pathway of alternative splicing (AS, orange) is able to generate alternatively spliced isoforms, which in turn can be translated into protein isoforms in response to hypoxia treatment. In the third pathway of alternative translation initiation (ATI, violet), upstream ORFs (uORFs) and small ORFs (sORFs) can further expand the protein diversity under hypoxia treatment.

In conclusion, this study expands our understanding of the genome coding ability of rice under hypoxic germination. Two post-transcriptional mechanisms, AS and ATI, make major contributions to protein diversity during hypoxia (Fig. 8). AS may function in parallel with transcriptional control (e.g. ERFVII transcription factors) in response to hypoxia stress during rice germination. Specifically, low oxygen conditions extensively affect AS and ATI patterns in parallel with conventional transcriptional regulation during rice germination. The compositional change of spliceosomes, especially for splicing factors, may result in the preferred usage of non-canonical splicing sites under hypoxia treatment. In this case, the conservation of 5'-splicing sites was greatly affected by the hypoxia treatment. Furthermore, hypoxia-affected DAS events were more likely to undergo protein translation in comparison with AS events identified under normal conditions. In addition, sORFs and novel frame proteins generated by ATI further expand the regulatory complexity and protein diversity of rice seeds in response to hypoxia, respectively. The above results indicate the existence of a large underground network of hypoxia responses at the post-transcriptional level. This newly discovered underlying response mechanism is mediated by AS and ATI. The members of this network need to be further characterized. This case study using hypoxic germination as a model demonstrates how modern technology and bioinformatic analysis improve our understanding of the plant genome coding ability and its features during stress responses.

## Supplementary data

Supplementary data are available at *JXB* online.

Fig. S1. Analytical pipeline of AS identification, quantification, and validation in this study.

Fig. S2. Phenotypic characterization of rice seed germination under hypoxia.

Fig. S3. Biochemical indicators of rice seeds during hypoxia germination.

Fig. S4. Comparison of previous published data sets and qRT-PCR validation.

Fig. S5. GO enrichment analysis between DAS and DEG data sets from RNA-sequencing.

Fig. S6. qRT-PCR validation of selected genes from DAS events.

Fig. S7. Comparison of splicing site recognition between AS and DAS events.

Table S1. Summary of the basic parameters in the RNA sequencing data set.

Table S2. List of differentially expressed genes.

Table S3. List of the differentially expressed AS events.

Table S4. Summary of quantified proteins in proteomic analysis.

Table S5. List of the differentially expressed AS events and proteins of seed storage proteins.

Table S6. Prediction of subcellular localization of splicing isoforms.

Table S7. List of frame proteins and their location on corresponding mRNAs in air control samples.

Table S8. List of frame proteins and their location on corresponding mRNAs in hypoxia-treated samples.

Table S9. Primers used in this study.

Supplemental_Tables

Supplemental Figures

## Author contributions

MXC, FYZ, JHZ, and YGL designed the experiments. MXC, FYZ, FZW, NHY, TF, YYC, TYL, XZ, and SSZ performed experiments. MXC, FYZ, BG, KLM, GYF, ZZS, LJX, QJH, and HJW analysed the data. FYZ, MXC, and NHY wrote the manuscript. SX, JHZ, and YGL. critically commented on and revised the manuscript.
