# Supplementary material for: Alternative splicing and translation play important roles in hypoxic germination in rice
Source: J Exp Bot. 2018 Dec 10;70(3):817–33. doi: 10.1093/jxb/ery393 (PMC6363088; doi:10.1093/jxb/ery393)
Supplement: Supplemental Figures [file ery393_suppl_supplemental-figures-s1-s7.pdf]

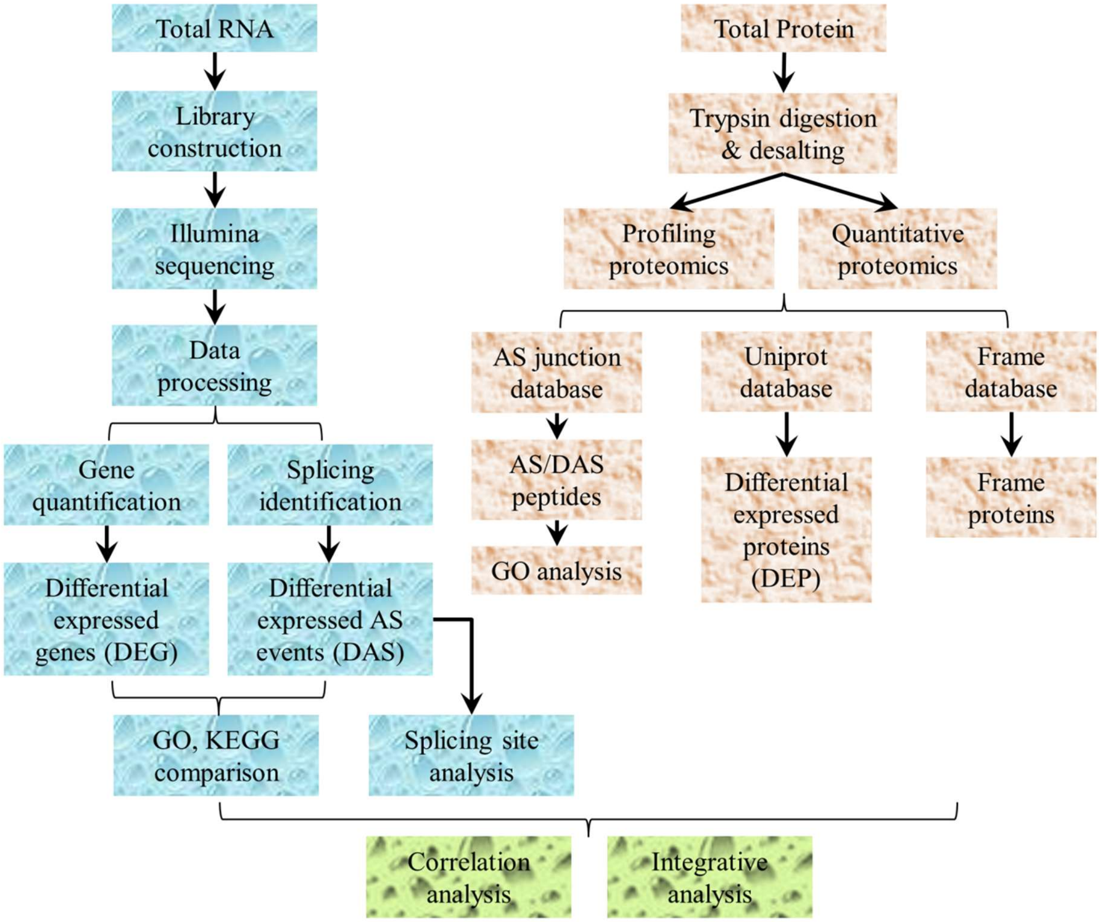

Figure S1 Analytical pipeline of AS identification, quantification and validation in this study.

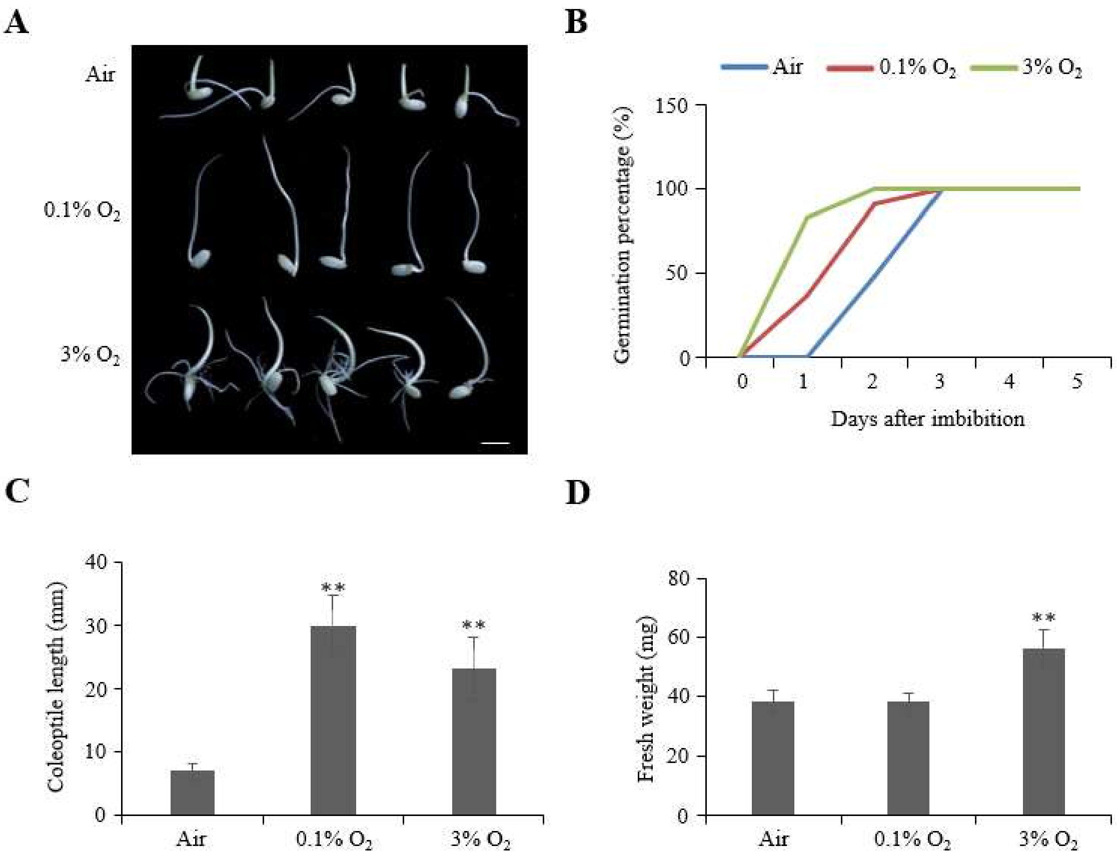

Figure S2 Phenotypic characterization of rice seed germination under hypoxia. (A) Comparison of phenotypes of 7-day rice seedlings under 1% O_2 and 3% O_2 treatment versus air control. (B) Representation of germination curves of aforementioned three treatment groups. Three biological replicates (3 petri dishes, with 30-50 seeds per petri dish) were taken to calculate germination percentage from day 1 to day 5 at 24h interval. Measurement of coleoptile length (C) and fresh weight of rice seedlings subjected to hypoxia treatment (1% O_2 or 3% O_2) or air control on day 7. Values are means ± SE, (n=30 to 45 for coleoptile length measurement, n=10 to 12 for fresh weight measurement). The '**' denotes that the value is significantly higher in hypoxia-treated samples in comparison to air control by Student's *t*-test (P<0.01).

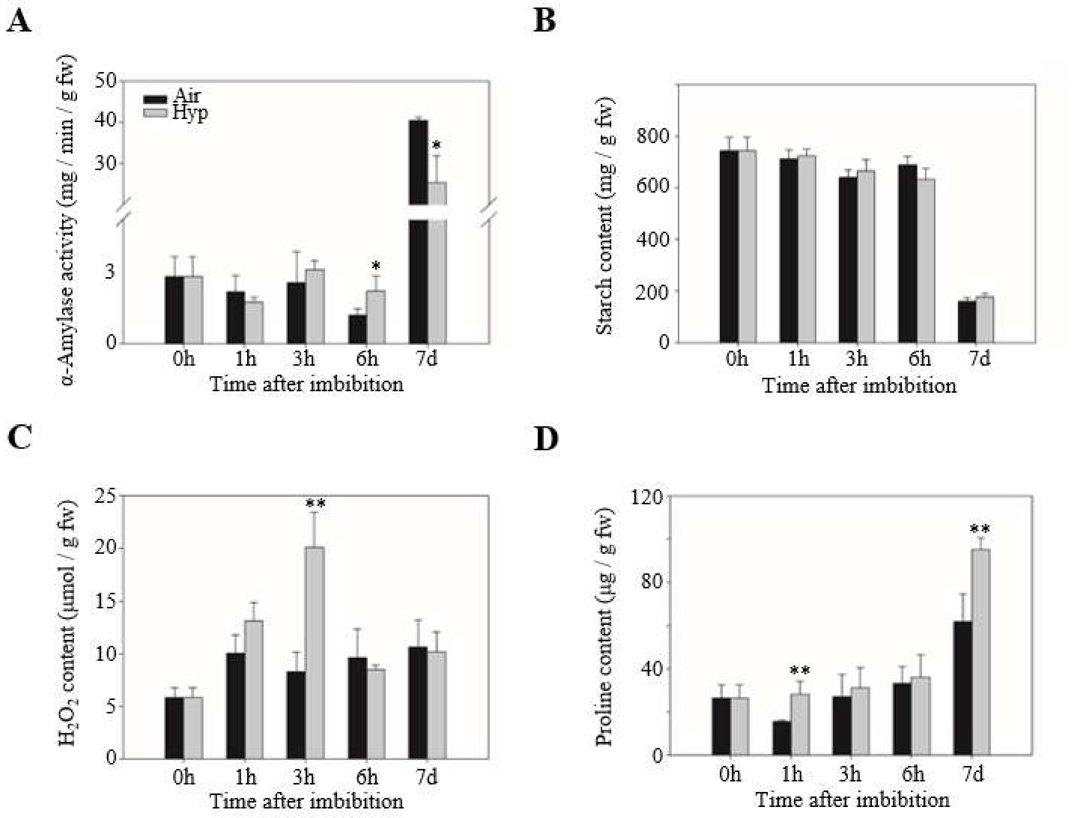

Figure S3 Biochemical indicators of rice seeds during hypoxia germination. Biochemical parameters including (A) α-Amylase activity, (B) starch content, (C) H_2O_2 contnt, (D) proline content, were measured at 0h, 1h, 3h, 6h and 7d after hypoxia treatment (3% O_2) in comparison to air control. Values are means ± SE, (n=4). The '*' and '**' denote that the value is significantly higher or lower in hypoxia-treated samples (grey bars) in comparison to air control (black bars) by Student's *t*-test (P<0.05 and P<0.01), respectively.

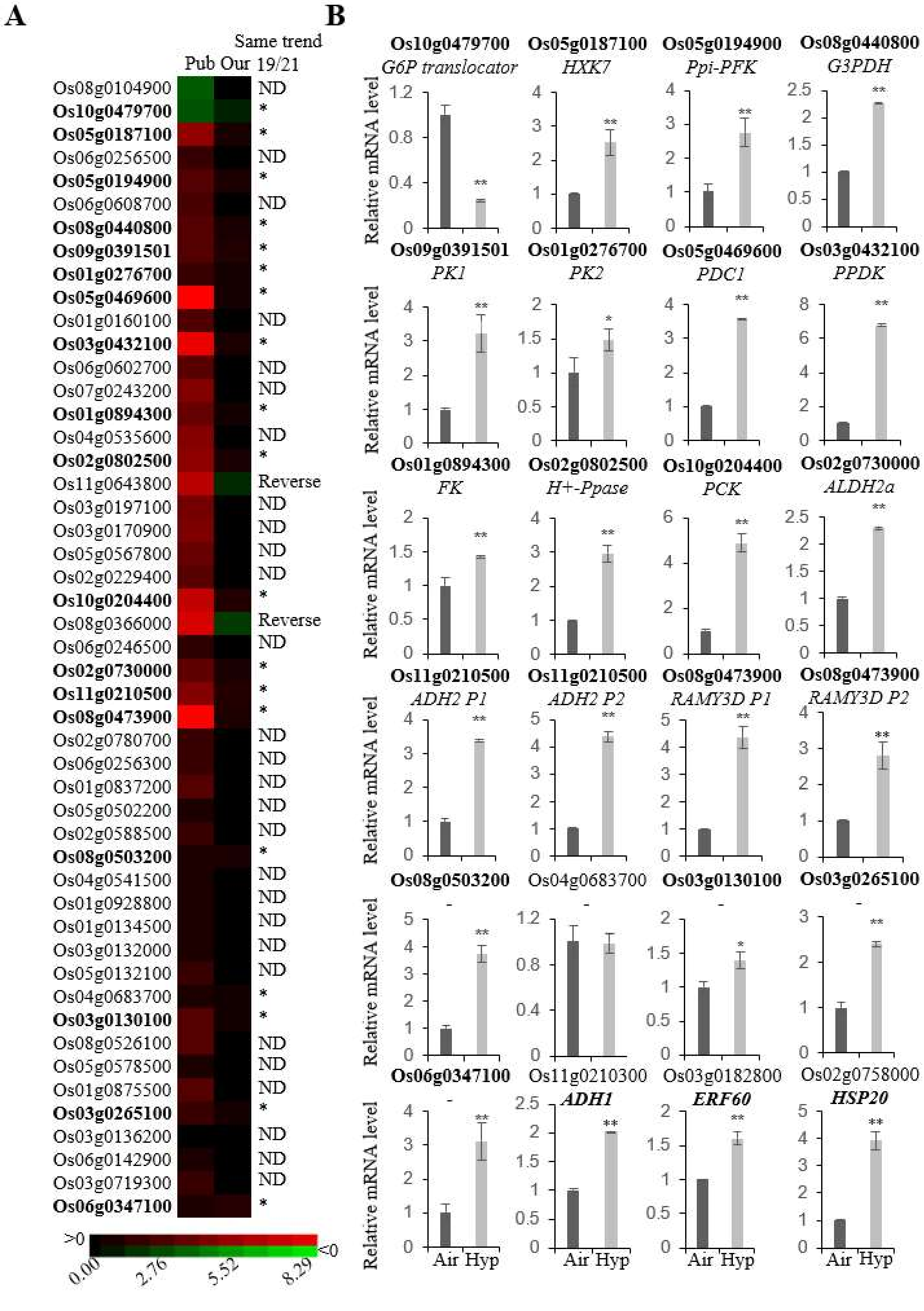

Figure S4 Comparison of previous published datasets and qRT PCR validation. (A) Heatmap comparison of previous published microarray datasets (Pub) to our RNA seq analysis (Our). (B) qRT-PCR validations of the selected and marker genes during hypoxic germination from three biological replicates. *OsACTIN1* was used as an internal reference gene. ‘*’ and ‘**’ denote that the

relative mRNA level is significantly higher in hypoxia-treated samples (grey bars) in comparison to air control (black bars) in complete darkness, $P<0.05$ and $P<0.01$, respectively. **Locus IDs (Bolded)** represent genes have similar expression pattern in qRT-PCR analysis in comparison to previous transcriptome or microarray analysis.

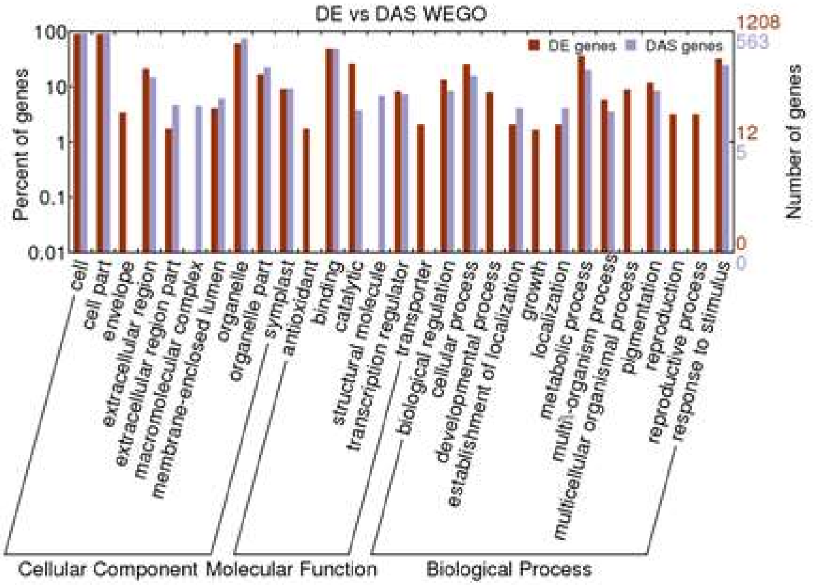

Figure S5 GO enrichment analysis between DAS and DEG datasets from RNA sequencing.

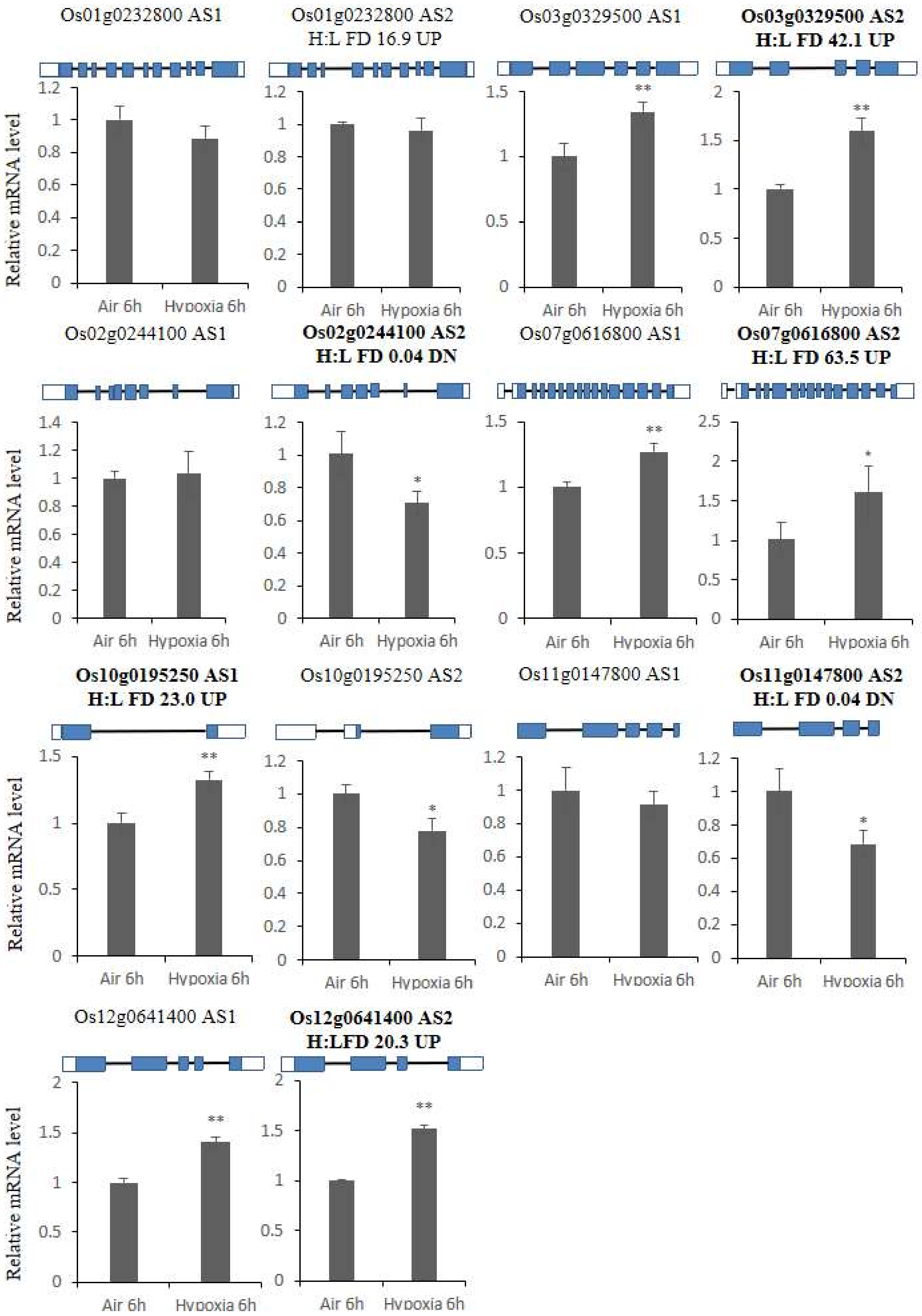

Figure S6 qRT-PCR validation of selected genes from DAS events. Primers used in the experiment are listed in Supplemental Table 9. *OsACTIN1* was used as an internal reference gene. '*' and '**' denote that the relative mRNA level is significantly higher or lower in hypoxia-treated samples in comparison to air control, $P<0.05$ and $P<0.01$, respectively. AS events in bold form represent the consistency between RNA seq and qRT-PCR data.

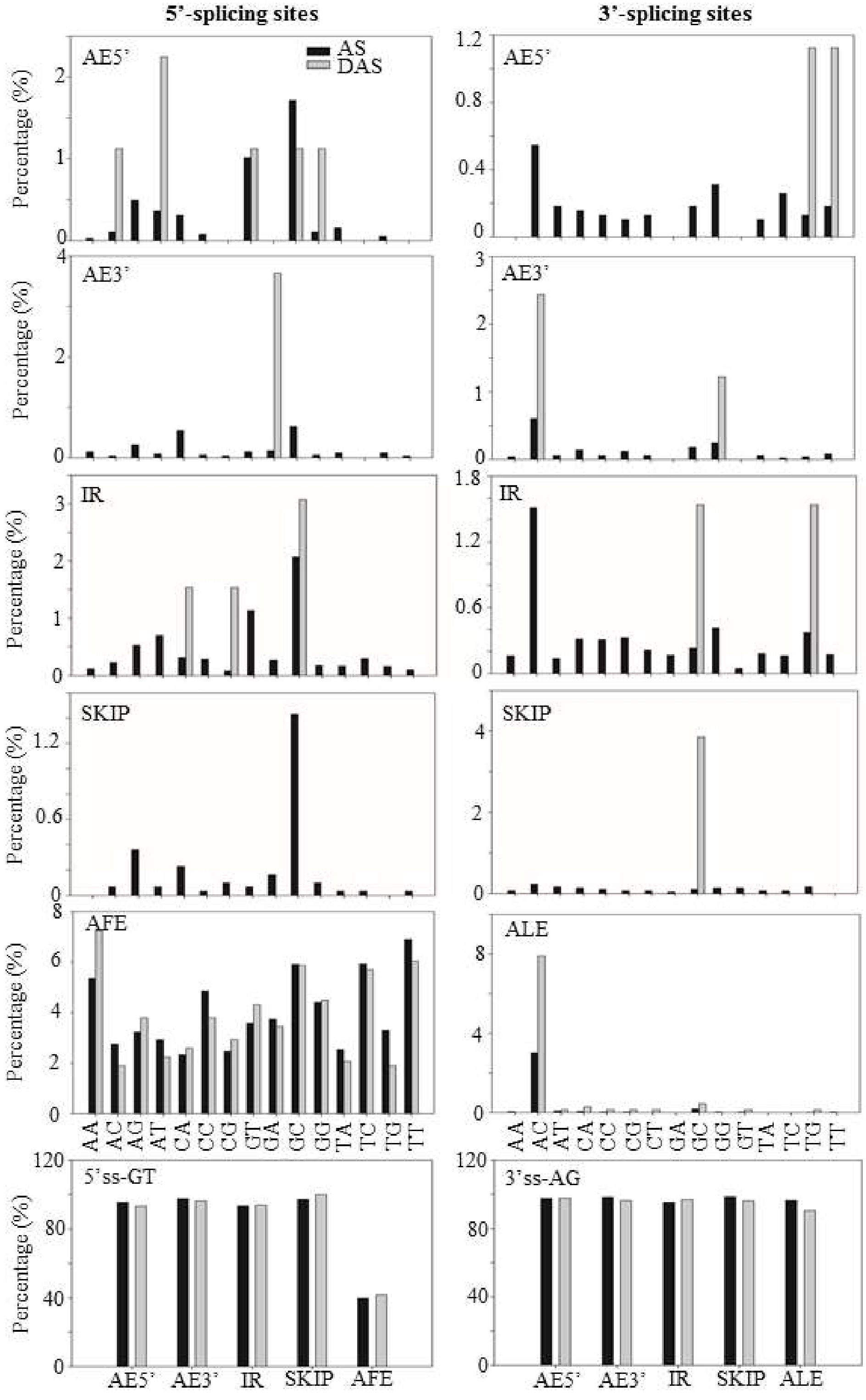

Figure S7 Comparison of splicing sites (ss) recognition between AS and DAS events. ALE, alternative last exon; AFE, alternative first exon; SKIP, exon skipping; IR, intron retention; AE5', alternative donor; AE3', alternative acceptor.
